# HIV serostatus disclosure among people living with HIV/AIDS in Mwanza, Tanzania

**DOI:** 10.1186/1742-6405-11-5

**Published:** 2014-01-22

**Authors:** Gladys Yonah, Francis Fredrick, Germana Leyna

**Affiliations:** 1 Bombo Regional Hospital, Tanga, Tanzania; 2 Department of Paediatrics, School of Medicine, Muhimbili University of Health and Allied Sciences, Dar es Salaam, Tanzania; 3 Department of Epidemiology and Biostatistics, School of Public Health and Social Sciences, Muhimbili University of Health and Allied Sciences, Dar es Salaam, Tanzania

## Abstract

**Background:**

Disclosing HIV serostatus is important for HIV prevention and maintenance of health for people living with HIV their spouses and the community, it plays a role in the social relation which is critical in reducing HIV transmission. The process may have positive and negative effects to the HIV infected people who disclose their status. The present study was undertaken to describe HIV serostatus disclosure among HIV infected people attending care and treatment clinic at Sekou-Toure hospital in Mwanza, Tanzania.

**Methods:**

A cross-sectional study was carried out on 270 HIV infected adults attending Care and Treatment Clinic (CTC) at Sekou-Toure hospital between September and October, 2010. A Swahili questionnaire was used to obtain demographic and HIV disclosure information.

**Results:**

Hundred and ninety five (72.5%) of all recruited participants were females, 88.1% (238/270) were aged above 30 years and 44.1% (119/270) were married. The prevalence of serostatus disclosure was 93.3% (252/270) with participants aged above 30 years having significantly higher proportion of serostatus disclosure compared to those aged below 30 years (94.5% vs. 84.4%, p < 0.05). Among the participants who disclosed their status, 69.3% reported closeness to the disclosed person as the reason for disclosure while 25.8% (65/252) disclosed because they needed help. Two hundred (79.4%) reported to have received emotional support following disclosure while 25.8% and 29.7% received financial support and freedom to use their anti-retroviral drugs around the person they disclosed their status respectively. Thirty four participants reported to have been discriminated following disclosure and 12 participants reported to have been divorced.

**Conclusions:**

Rate of disclosure of HIV serostatus was noted to be high in this study. Delayed disclosure was also noted in small proportion of participants. Negative outcomes following disclosure of serostatus were reported by participants. Efforts need to be increased to promote disclosure of HIV serostatus in Tanzania through health education and awareness for both HIV infected individuals and the community.

## Background

HIV/AIDS is still a major burden to the health system in Tanzania as it is for other Sub-Sahara African countries. The current estimate for HIV prevalence in Tanzania is 5.6% [1] it is about 1.3% lower than previously reported rate [2]. It is estimated that 1.4 million people are living with HIV in Tanzania [1] with females more affected as compared to their male counterparts.

Prevention of new infection is the cornerstone in the fight against HIV/AIDS. Disclosure of HIV status is an important approach pursued in voluntary testing and counselling (VCT) services. Maman reported 64.5% disclosure among HIV positive women attending VCT clinic in Dar es Salaam-Tanzania [3]. In another study conducted among HIV infected pregnant women in Morogoro, Tanzania 41% had disclosed to their partners [4].

Studies conducted in Tanzania have reported age, level of education and financial independence particularly for women to be important factors in predicting HIV serostatus disclosure. Kiula et al. in a study conducted in Morogoro, Tanzania reported that HIV-positive women with high level of education, younger and financially independent to be more likely to disclose their serostatus [4]. Lugalla et al. reported women to be less likely to reveal their status to their spouses as compared to men in a study which was conducted among clients attending VCT in Tanzania [5]. In a study conducted by Mwanga et al. among people living with HIV attending care and treatment clinic in Kisarawe, Tanzania disclosure was noted to be similar between males (97.0%) and females (98.5%), however male participants (74.2%) were noted to disclose more to their spouses than female participants (47.7%) [6].

HIV status disclosure has been reported to benefit PLWHA in several ways including psychological, emotional and material support from family and other community members and freedom to use ARV medications [7-9]. HIV transmission occurs between partners including spouses, therefore disclosure of status will provide timely access to care and treatment services in case the partner/spouse is also affected [10]. Uninfected partners in discordant couples will benefit through taking appropriate actions including safer sex practices to prevent acquisition of HIV. Negative outcomes have been attributed to disclosure; these may include rejection, assault, separation, divorce, stigma and discrimination [3,8]. This study was conducted to examine reasons and effects of HIV serostatus disclosure among PLWHA attending CTC clinic at Sekou-Toure Hospital in Mwanza, Tanzania.

## Methods

### Study design and population

A hospital based cross-sectional study was conducted between September and October, 2010 at Sekou- Toure hospital in Mwanza, Tanzania. A total of 270 (89%) adults living with HIV/AIDS aged 18 years and above who were attending care and treatment clinic for more than six months were recruited. The sample size was determined using proportional formula for calculating sample size; a prevalence of disclosure of 23% from Zimbabwe [11] was utilized for sample size calculation aiming at a power of 80% with a response rate of 90%.

### Study area

Mwanza is the second largest city in Tanzania located on the Southern shore of Lake Victoria with an estimated population of 2.5 million inhabitants. Sekou-Toure is a regional hospital serving as a referral hospital to five district hospitals. The care and treatment clinic is specific clinic offering services exclusively to HIV infected patients. The clinic is operating daily on working days and it is manned with two clinicians and four nurses. The average patients’ attendance is 40 patients in each clinic day.

### Data collection

Data were collected using pre-tested interviewer-administered Swahili questionnaires which were developed for this study. Participants were recruited consecutively at the CTC clinic during their monthly visits. All consented participants were interviewed in the clinic. Participants were requested to participate voluntarily and no compensation was provided for this study. The information sought included demographic variables which were age, sex, marital status, level of education and occupation. Other information sought from participants included HIV disclosure status, time of disclosure, outcomes following disclosure and reasons for and for not disclosing. Single multiple response questions were asked to determine disclosure status: “Have you ever told anyone about your HIV status?” The person disclosed to was determined by asking: “who was the first person you disclosed your HIV status to?” If the individual had not disclosed his/her HIV status the reasons for not disclosing were explored. Immediate reaction of person disclosed to were probed and the perceived benefits and adverse outcomes were explored from participants. Reasons for not disclosing and outcomes following disclosure were expressed as frequencies according to participants’ responses, more than one response was provided by some participants. Participants were asked if they felt stigmatized and discriminated following disclosure.

### Ethical considerations

This study was approved by the MUHAS ethical review committee. Permission to carry out the study was sought from district health authorities and the hospital management. Participants gave verbal informed consents prior to recruitment into this study.

### Data analysis

Epi Info version 6 statistical package was used for data entering, cleaning and analysis. Chi square and Fischer’s exact test were used to determine association between categorical variables. A probability (p) value of ≤0.05 was considered statistically significant.

## Results

A total of 270 (89%) participants were recruited during the study duration. Of the participants 72.2% (195/270) were females and 88.1% (238/270) were aged above 30 years, the mean age of study participants was 39.3 ± 8.5 years. One hundred and nineteen (44.1%) were married, 73.7% (199/270) had primary school education and 73.0% (197/270) were employed. Two hundred and fifty two participants (93.3%) had disclosed their HIV sero-status. As shown in Table 1, male and female had similar disclosure status 93.3%. The difference between participants who disclosed and did not were not significant except for age category whereby the proportion of participants who had disclosed their status was higher among participants aged above 30 years as compared to those aged below 30 years (94.5% vs. 84.4%, p = 0.0304) as shown in Table 1. Fifty percent of participants who disclosed reported to have done it to their family member/close relative while 25.4% disclosed to their spouses and 19% to their parents/guardians as described in Table 2.

**Table 1:** Prevalence of disclosure status by demographic characteristics among PLWHA (N = 270)

			**Disclosure status**		
**Socio-demographic characteristic**	**Category**	**N**	**Yes n (%)**	**No n (%)**	**P-value**
**Sex**	Females	195	182 (93.3)	13 (6.7)	0.9777
	Males	75	70 (93.3)	5 (6.7)	
**Age**	< 30 years	32	27 (84.4)	5 (15.6)	0.0304
	> 30 years	238	225 (94.5)	13 (5.5)	
**Education**	Informal	43	40 (93.0)	3 (7.0)	0.6679
	Primary	199	186 (93.5)	13 (6.5)	
	Secondary	26	24 (92.3)	2 (7.7)	
	College/university	2	2 (100)	0 (0)	
**Marital status**	Single	9	8 (88.9)	1 (11.1)	0.5845
	Married	119	111 (93.3)	8 (6.7)	
	Cohabiting	32	28 (87.5)	4 (12.5)	
	Widow(er)	63	60 (95.2)	3 (4.8)	
	Divorced	47	45 (95.7)	2 (4.3)	
**Occupation**	Employed	197	183 (92.9)	14 (7.1)	0.6679
	Self-employed	33	32 (97.0)	1 (3.0)	
	No specific job	40	37 (92.5)	3 (7.5)	

**Table 2:** Distribution of recipients of disclosure and their initial reactions

**Variable**	**Number (%)**
*To whom did you disclose your HIV status first? (N = 252)*	
Spouse	64 (25.4%)
Parent/guardian	48 (19.0%)
Close relative	128 (50.8%)
Close friend	10 (4.0%)
Others	2 (0.8%)
*What was the reaction of the first person to whom you disclosed your status?(n = 252)†*	
She/he was shocked	41 (16.3%)
She/he cried	7 (2.8%)
She/he was understanding	137 (54.4%)
She/he laughed at me	1 (0.4%)

† 66 participants did not report any reaction.

Majority of participants 169/252 reported to have disclosed their status because of close relationship to the person they told. Other reasons for disclosing included need for help (65/252) and advice from VCT care providers (28/252). Two hundred and fourteen participants (84.9%) disclosed their status within one month of diagnosis, 14/252 (5.6%) between one to six months and 24/252 (9.5%) disclosed after six months of knowing their HIV status. Reasons given by 18 participants who did not disclose their status included fear of being abandoned by family members reported by six participants, fear of divorce and abandonment by family members (2/18), fear of divorce was reported by only one participant (1/18). Five participants reported that they did not see the importance of disclosing while four did not give any reason.

Emotional support following disclosure was reported by 124 (49.2%) while financial support following disclosure was reported by 11.9% (30/252) of participants. Some participants (13.1%) reported that they received emotional support, financial support and were able to use their ARV freely following disclosure as opposed to the habit of hiding their medications and using them secretly which was the case before disclosure,. The negative effects of disclosure reported in this study included stigma and discrimination which was reported by 13.5% (34/252) and divorce which resulted from disclosure as reported by 10% (12/111) of married participants (Table 3).

**Table 3:** HIV status disclosure outcome

**Experienced outcomes**	**n**	**%**
***Positive outcomes (n = 252)***		
Emotional support	124	49.2%
Freedom to use ART	12	4.8%
Financial support	30	11.9%
Freedom to use ART and emotional support	28	11.1%
Freedom to use ART and financial support	2	0.8%
Financial support, freedom to use ART and emotional support	33	13.1%
***Negative outcomes***		
Stigma and discrimination (n = 252)	34	13.5%
Divorce (n = 111)†	12	10.8%

†n is the total number of married participants who disclosed their serostatus.

## Discussion

This study was conducted to determine reasons and effects of HIV serostatus disclosure among PLWHA in Mwanza, Tanzania. Most people living with HIV/AIDS (93.3%) reported to have disclosed their serostatus. Findings from this study are similar to reports by Deribe et al. (94.5%) and Seid et al. (93.1%) from studies conducted among HIV infected men and women using clinical services in Ethiopia [12,13]. The rate of disclosure was similar between males and females in this study, contrary to reports by Anglewicza et al. and Simbayi et al. in studies conducted in Malawi and South Africa respectively, which noted higher rates of disclosure to sexual partners among female participants as compared to males [14,15]. Mwanga et al. reported higher proportion of male participants to have disclosed to their sexual partners in a study conducted among CTC clients in Kisarawe district in Tanzania [6].

Participants aged above 30 years had significantly higher prevalence of serostatus disclosure as compared to those aged below 30 years. Similar findings were reported by Maman et al. and Zou et al. both of which found older age to be a predictor of HIV serostatus disclosure, both studies were carried out in Tanzania [7,16]. Zou et al. reported older Christians to be more willing to disclose their serostatus if they were found to be HIV positive. Contrary to these findings Kiula et al. reported younger pregnant women to be more likely to disclose their serostatus in a study conducted in Morogoro, Tanzania [4]. The likelihood of disclosure among older participants in our study may be attributed to their maturity and sense of responsibility; however there seem to be a need to explore in depth on the influence of age on HIV serostatus disclosure in Tanzania.

Disclosure to close relative was reported by half of the participants who told their status and only 25% reported spouse as the first person they told their status. This is contrary to report by Lugalla et al. who reported 42% of respondents to have disclosed to their spouses in a study which was conducted among CTC clients who were recruited after completion of testing and counselling in Tanzania [5]. It is possible that married participants in our study disclosed their status to their spouses subsequently; however any delays could have serious implication in the transmission of HIV particularly for discordant couples in events where protective measures like condoms would have not been used during sexual intercourse.

It is encouraging that majority (84.9%) of participants disclosed within six months of knowing their diagnosis. However 15.1% of participants had delayed disclosure similar to 14.2% reported by Deribe et al. in a study conducted among HIV infected men and women using clinical services in Ethiopia [12]. Lugalla et al. reported lower rates of disclosure within the first month of diagnosis in a study conducted among VCT clients in Tanzania in which more than a quarter of the respondents reported disclosure within one month of diagnosis [5]. Delayed disclosure increases the risk of transmission of HIV infections especially when condoms are not used during sexual contact and may downplay the benefits of HIV status disclosure, this may be particularly the case immediately following the receipt of HIV results.

Serostatus disclosure plays an important role in improving quality of life for PLWHA, benefits of serostatus disclosure reported in this study included freedom to use ARV drugs, financial and moral support, similar findings have been reported in literature, Stirratt et al. in a study which was conducted in New York among HIV seropositive patients reported poor adherence to ART among participants who had not disclosed their status [17]. Norman et al. reported financial and emotional support following disclosure from family and community members in a study conducted among PMTCT clients and health care providers in South Africa [7]. Advice and counselling from health care providers were reported to have led to HIV serostatus disclosure among our participants, this finding demonstrate the important role played by health care providers in facilitating HIV status disclosure. Role of health care providers in facilitating disclosure have been previously reported, Kadowa et al. and Mucheto et al. reported counselling and advice from health care providers to predict disclosure in studies conducted among PLWHA in Uganda and Zimbabwe respectively [7,18-21].

Negative outcomes following HIV serostatus disclosure may result in individuals opting to withhold their status to family, friends and community. Stigma, discrimination and divorce following HIV sero-status disclosure was reported by few participants in this study. This signifies the importance of increasing awareness in our communities to alleviate stigma attached to HIV infection. Separation and divorce have been documented as important factor hindering disclosure, Mucheto et al. reported women who believed disclosure would cause divorce were to be less likely to disclose in a study conducted among HIV infected women attending antenatal and postnatal clinic in Zimbabwe [7,19,22]. Couple counselling may be a useful strategy to mitigate spousal rejection and enhance serostatus disclosure in our communities.

### Study limitations

This study was based on self-reporting which may have over or underestimated the disclosure status. This was a cross-sectional study therefore some aspects of disclosure could not have been explored adequately. Findings of this study may not be generalizable to the community setting as it was carried out in one health facility.

## Conclusions

High rate of HIV disclosure noted in this study is encouraging, however it is important to consider the negative outcomes following disclosure reported in this study. The delay in disclosure and the small proportion of participants who had not disclosed sends an important message to the players in the fights against HIV/AIDS to put emphasis on increasing awareness in the community on the impact of stigma and discrimination. There is a need therefore, to explore factors which are hindering successful and timely disclosure of HIV serostatus in Tanzania.

## Competing interests

Authors declare that there are no competing interests.

## Authors’ contributions

GY - designed the study, prepared questionnaire, collected data, performed preliminary analysis and wrote the preliminary report for this study. GL - designing of the study, data collection, data analysis and review of manuscript. FF - participated in data analysis and drafting first manuscript for this study. All authors have read and approved this manuscript.
